# Cellular signaling impacts upon GABAergic cortical interneuron development

**DOI:** 10.3389/fnins.2023.1138653

**Published:** 2023-03-14

**Authors:** Emily Ling-Lin Pai, April M. Stafford, Daniel Vogt

**Affiliations:** ^1^Department of Pathology and Laboratory Medicine, Hospital of the University of Pennsylvania, Philadelphia, PA, United States; ^2^Department of Pediatrics and Human Development, Michigan State University, Grand Rapids, MI, United States; ^3^Neuroscience Program, Michigan State University, East Lansing, MI, United States

**Keywords:** mTOR, MAPK, Wnt/beta-catenin, cortical interneuron, cellular signaling

## Abstract

The development and maturation of cortical GABAergic interneurons has been extensively studied, with much focus on nuclear regulation *via* transcription factors. While these seminal events are critical for the establishment of interneuron developmental milestones, recent studies on cellular signaling cascades have begun to elucidate some potential contributions of cell signaling during development. Here, we review studies underlying three broad signaling families, mTOR, MAPK, and Wnt/beta-catenin in cortical interneuron development. Notably, each pathway harbors signaling factors that regulate a breadth of interneuron developmental milestones and properties. Together, these events may work in conjunction with transcriptional mechanisms and other events to direct the complex diversity that emerges during cortical interneuron development and maturation.

## Introduction

The processes by which cortical GABAergic interneurons (CINs) develop, differentiate and acquire their unique properties have been studied for many years. Insights into the transcriptional cascades that guide these events have been well investigated, especially in early developmental stages, when CIN progenitors are first programmed and start to diverge into unique trajectories. In recent years, further stages during development and maturation have been explored, including how the local environment impacts their molecular and electrophysiological properties. Moreover, cellular signaling events may be involved in facilitating transcription factor recruitment, linking extracellular cues, and orchestrating neuronal activity, which may be translated downstream into nuclear events or other signaling intermediates.

Multiple ventral telencephalic regions, including the medial ganglionic eminence (MGE), caudal ganglionic eminence (CGE), and preoptic area (POA) generate CINs (Wonders and Anderson, 2006; Miyoshi et al., 2010; Gelman et al., 2011). The production, initial development and roles of CINs in brain disorders have been reviewed elsewhere (Marín, 2012; Hu et al., 2017; Wamsley and Fishell, 2017; Lim et al., 2018) and will not be covered extensively here. The MGE gives rise to ∼70% of CINs, with roughly equal numbers expressing parvalbumin (PV) or somatostatin (SST). The CGE gives rise to ∼20% of CINs that broadly express the 5HT3A receptor (Rudy et al., 2011), which can be subdivided into groups positive for vasoactive intestinal peptide (VIP) and neurogliaform cells that express markers such as REELIN and NDNF (Miyoshi and Fishell, 2011; Abs et al., 2018). Importantly, while these broad groups of CINs have common markers, they can also be subdivided into more refined classes, suggesting the presence of multiple CIN types that together drive brain inhibition.

How these diverse properties arise has been a massive endeavor in the field, many decades of which determined transcriptional codes necessary to drive development of diverse CIN lineages. Ventral telencephalon identity is initially formed *via* a sonic hedgehog (SHH) morphogen gradient, which induces the expression of transcription factors (TFs) such as *Nkx2.1*, *Dlx2*, and *Gsx* that promote the early development of MGE and CGE (Xu et al., 2005, 2008, 2010). In the MGE, initial patterning is determined by the transcription factor, *Nkx2.1*; its loss results in an expansion of adjacent ganglionic eminences (Sussel et al., 1999). Importantly, *Nkx2.1* is necessary for *Lhx6* expression (Du et al., 2008), a cardinal TF required for the emergence of both PV and SST lineagess (Liodis et al., 2007; Zhao et al., 2008). While not required for initial MGE patterning, loss of *Lhx6* leads to CINs with CGE molecular and electrophysiological properties (Vogt et al., 2014), suggesting some role in regional cell fate determination. To date, a cardinal TF that drives CGE patterning, like *Nkx2.1* for MGE, has not been discovered for CGE. However, the TFs *Gsx1 and 2* are important for CGE development (Corbin et al., 2000; Toresson et al., 2000; Yun et al., 2001; Xu et al., 2010; Wang et al., 2013), even though no patterning equivalent to *Nkx2.1* exists for the CGE. Some other genes like *Prox1* and *Nr2f1 and 2* also contribute to CGE-lineage CIN properties (Miyoshi et al., 2015; Touzot et al., 2016).

While TF cascades are necessary for many CIN developmental programs, it is unlikely they are the sole driver of CIN cell fate and function. In recent years, the role of the local environment and neural activity upon CINs has arisen as a potential candidate in either directing and/or refining the development and maturation of these diverse cells (Patz et al., 2004; De Marco García et al., 2011; Close et al., 2012; Denaxa et al., 2012; Dehorter et al., 2015; Wamsley and Fishell, 2017). Other studies have begun to suggest that cellular signaling arising in cytoplasmic compartments may also have a prominent role in CIN development and cellular properties (Vogt et al., 2015; Malik et al., 2019; McKenzie et al., 2019; Angara et al., 2020; Wundrach et al., 2020). Herein, we review these recent findings in the context of TF studies and suggest potential ways in which these observations may work together during CIN development and maturation.

## Transcription factor overview

Seminal studies on CIN cell fate and development have primarily investigated core TFs expressed in unique and overlapping patterns in the MGE, CGE and adjacent regions (Sussel et al., 1999; Zhao et al., 2008; Long et al., 2009; Flandin et al., 2011; Gelman et al., 2011). While these will not be covered in detail here, many of these TFs have been attributed to various CIN developmental milestones, including emergence of unique molecular, cellular and electrophysiological properties (Wonders and Anderson, 2006; Miyoshi et al., 2010; Kessaris et al., 2014; Hu et al., 2017; Wamsley and Fishell, 2017).

**FIGURE 1:**
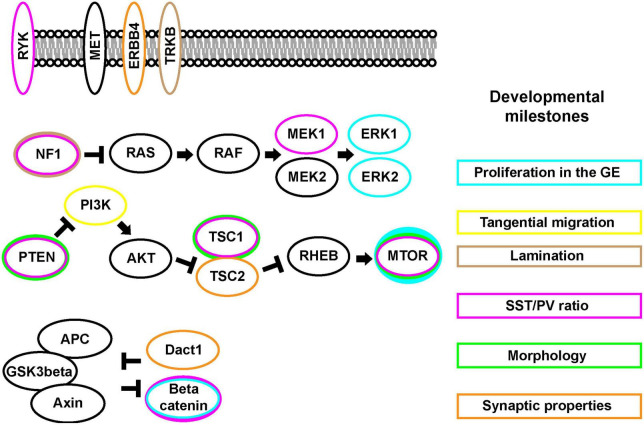
The mTOR, MAPK, and Wnt/Beta-catenin signaling pathways and CIN developmental events **(Right)**. CIN developmental key milestones **(Left)**. Signaling pathway proteins. Some of these signaling proteins are found at the cell membrane while many can be found in cytosolic compartments. Each protein may exhibit differential impacts on the CIN developmental milestones, colors around each protein match to the boxes around these events.

Within the MGE, *Nkx2.1* is a primary organizer of regional identity, while *Gsx1 and 2* contribute to properties of CGE development but not in the same manner as *Nkx2.1* in the MGE (Sussel et al., 1999; Corbin et al., 2000; Toresson et al., 2000; Yun et al., 2001; Xu et al., 2010; Wang et al., 2013). After these initial patterns are established, more restricted genes are expressed together or sequentially that determine the cardinal lineages of each region. *Lhx6* expression is necessary for the emergence of PV and SST expressing CINs (Liodis et al., 2007; Zhao et al., 2008). While less clear how CGE-lineage CINs are fated, TFs such as *Prox1* and *Nr2f1 and 2* have been implicated (Miyoshi et al., 2015; Touzot et al., 2016). These cardinal TFs further regulate subsequent TFs and regulatory genes, whose functions are attributed to CIN development and function. The MGE is the most studied region, with TFs such as *Sox6*, *Mafb/cMaf*, and *Satb1* genetically downstream of *Lhx6* that specify distinct aspects of CIN development (Zhao et al., 2008; Azim et al., 2009; Batista-Brito et al., 2009; Close et al., 2012; Denaxa et al., 2012; Vogt et al., 2014; Pai et al., 2019, 2020). Whether they are subject to regulation from cellular signaling proteins is just beginning to be examined. The role of signaling proteins during CIN milestones is highlighted in Figure 1.

## mTOR

The mechanistic target of rapamycin (mTOR) signaling pathway is critical for cellular growth, proliferation and synaptic properties (Laplante and Sabatini, 2012; Lipton and Sahin, 2014). Activation of the mTOR pathway is generally initiated *via* growth factors binding to their respective receptor tyrosine kinase receptors, which transduce a signal for PI3-kinase to phosphorylate the kinase, AKT (Alessi et al., 1997). AKT then phosphorylates the protein encoded by *Tsc2*, Tuberin, leading to its inhibition (Manning et al., 2002; Potter et al., 2002). In the absence of AKT activity, Tuberin forms an obligate complex with Hamartin, the gene encoded by *Tsc1* and acts as a GTPase activating protein toward Rheb (Inoki et al., 2003), halting its function. Rheb activity results in mTOR activation (Patel et al., 2003). While this pathway has been well studied, the functional consequences of mTOR signaling in several cell types is still lacking, with only a few studies in CIN development.

Many mTOR pathway genes are ubiquitously expressed, thus, CINs are hypothesized to be impacted by this cascade. Earlier studies found that the TrkB receptor promoted CIN tangential migration and discovered that an upstream activator of the mTOR pathway, PI3-kinase, promoted tangential migration of CINs (Polleux et al., 2002). Another study showed that conditional loss of *Tsc1* using the pan GABAergic *Dlx5/6-Cre* led to a loss of GABAergic lineages and increased sensitivity to a seizure inducing drug (Fu et al., 2012); both activation of TrkB and loss of *Tsc* genes result in hyperactivity of mTOR signaling. Recently, AKT activity mediated by a class of protocadherin, was shown to regulate programmed CIN apoptosis necessary for the proper balance of excitation and inhibition in the brain (Carriere et al., 2020). Overall, these studies implicate key mTOR pathway players in CIN development.

Other studies have investigated the use of more restrictive genetic manipulation of the mTOR pathway. One approach examined the loss of *Pten*, which inhibits mTOR signaling, from MGE progenitor cells using *Nkx2.1-Cre*, which begins to express in cells including MGE progenitors (Xu et al., 2008). Similar to the loss of cells using pan GABAergic *Dlx5/6-Cre* to delete *Tsc1* (Fu et al., 2012), this study also found a reduction in *Nkx2.1-Cre*-lineages and concluded that these cells accumulated in the lateral cortex during tangential migration; many *Pten* deleted cells were SST + that underwent apoptosis, skewing the SST/PV ratio (Vogt et al., 2015). In line with these data, expression of an active PI3K mutant in *Nkx2.1-Cre* lineages also led to fewer CINs (D’Gama et al., 2017). Interestingly, another group deleted mTOR itself using *Nkx2.1-Cre* and also found less CINs (Ka et al., 2017), suggesting that mTOR signaling needs to be balanced for proper development.

Using a more refined approach, the mTOR pathway inhibitor, *Tsc1*, was deleted from MGE lineages using *SST-Cre* (Malik et al., 2019). Key molecular and electrophysiological phenotypes occurred in SST-lineages after loss of *Tsc1*, with these cells acquiring PV-like properties. These included the increased expression of PV and the fast-inactivating potassium channel, Kv3.1, as well as elevated fast-spiking properties (Malik et al., 2019). This latter study implied that mTOR signaling may have a role in establishing the distinct properties of SST and PV CINs that had not been appreciated before. Recently, a pathway was elucidated whereby the ErbB4 receptor in PV CINs inhibited Tuberin to locally elevate mTOR activity and translation of critical synaptic proteins (Bernard et al., 2022), exemplifying the range of this pathway during development.

Finally, studies have revealed the potential for faster physiological screening of human genetic variants associated with neurological disorders by taking advantage of the unique properties of CINs, the ability once transplanted into a host environment to disperse and integrate into the target microcircuit (Alvarez-Dolado et al., 2006; Bráz et al., 2012; Howard and Baraban, 2016). Using this approach, translational studies have been performed to link human genetic variation with CIN development and function. Human *PTEN* genetic variants were screened for their ability to complement mouse *Pten* loss of function phenotypes in an *in vivo* assay of CIN development. This approach showed that these gene variants were hypofunctional at promoting normal CIN development (Vogt et al., 2015). Further studies investigated human genetic variants in *TSC1*, exposing similar hypofunction during CIN development (Wundrach et al., 2020).

## MAPK

Like the mTOR signaling pathway, the RAS/MAPK signaling cascade has several proteins expressed ubiquitously, although some may be more enriched in CINs versus other neurons (Ryu et al., 2019). This cascade responds to various growth cues and neural activity and can signal to cytoplasmic targets as well as impact nuclear transcription, which in turn influences cell proliferation, maturation, survival and synaptic plasticity (Waltereit and Weller, 2003; Wiegert and Bading, 2011; Sun et al., 2015; Tyssowski et al., 2018). This cascade is also primarily made up of interacting GTPases and kinases that can be grouped into activators and inhibitors of the pathway that we will review; for more detailed description of the pathway, see (Seger and Krebs, 1995; Sun et al., 2015).

An early study implicated that GABAergic dysfunction underlies spatial memory deficits in an *Nf1* deletion model (Costa et al., 2002); using the same Cre line to express active KRAS had similar effects on behavior and increased GABAergic synapses (Papale et al., 2017). *Nf1* inhibits the enzymatic activity of the RAS GTPase in the initial stages of the MAPK pathway. Thus, *Nf1* loss results in elevated MAPK signaling. Two recent studies investigated the role of hyperactivating the MAPK pathway in more restricted *Nkx2.1-Cre* MGE lineages; loss of *Nf1* and expression of a constitutively active *Mek* allele, led to a roughly 50% reduction in PV expressing CINs in each model (Angara et al., 2020; Holter et al., 2021). These data uncovered a role for MAPK hyperactivity on the development of distinct classes of CINs. Importantly, the studies revealed common phenotypes in families of signaling proteins during CIN development, which could have implications for other MAPK syndromes and common comorbid diagnoses, including autism (Adviento et al., 2014).

While no studies have yet determined the role of the pathway on CGE lineages, there is some evidence for ERK1/2 signaling in CGE proliferation (Stanco et al., 2014). Moreover, a study revealed a role for MAPK hyperactivity on a cardinal MGE TF, *Lhx6*. Specifically, Loss of *Nf1* in *Nkx2.1-Cre* lineages resulted in a depletion of *Lhx6* transcript (Angara et al., 2020); this was also prominent in mutants that only lost one allele of *Nf1*, which is similar to humans diagnosed with Neurofibromatosis 1. Thus, in addition to altering PV expression in CINs, elevated MAPK signaling may influence the development of these cells *via* regulation of core cardinal TFs. While this study specifically examined *Lhx6* expression, other core programs that direct CIN development have not yet been assessed in an unbiased manner, which may lead to further key discoveries that MAPK signaling impinges upon.

In addition to the RAS/MAPK pathway that involves RAF/MEK/ERK signaling there are other MAPK signaling cascades that may impact CIN development. One example comes from the parallel MAPK signaling cascade that transduces signals through the JNK proteins. Initial studies have identified JNK1 as necessary for proper CIN tangential migration (Myers et al., 2014) and further studies suggested that laminar position of CINs could also be regulated by JNK dysfunction (Myers et al., 2020).

## Wnt/beta-catenin

While less studied in CIN development there have been inroads made into canonical wingless-related integration site (Wnt)/beta-catenin and related non-canonical signaling events. This pathway has early effects on MGE progenitors *via* canonical Wnt/beta-catenin signaling, as loss of beta-catenin resulted in less proliferating MGE cells (Gulacsi and Anderson, 2008). One examined the role of the Wnt/beta-catenin scaffold gene, *Dact1*, and found an impact on forebrain synapse development (Arguello et al., 2013).

WNT ligands are also enriched closer to caudal regions of the MGE and the MGE responds in turn to WNT signaling in these regions, which predominantly generate SST + CINs (McKenzie et al., 2019). While this study did not find a significant role for canonical WNT signaling in the promotion of SST CINs, non-canonical signaling *via* the RYK receptor emerged as a potential moderator of SST expression in some caudal MGE populations when active RYK protein signaling was modulated. However, RYK’s role during development is complicated, as loss of function in MGE progenitors leads to several unidentified lineages with no observable ratio change in SST and PV CINs, suggesting a further role in MGE progenitors. Moving forward, this story may help bridge other work on *Ryk* and CINs (Chang et al., 2017).

The TFs *Mafb* and *c-Maf* are involved in GSK3-beta signaling; phosphorylation of MAF proteins by GSK3-beta leads to increased protein activity downstream of MAFs (Rocques et al., 2007; Herath et al., 2014). Deletion of *Mafb* and *c-Maf* using *Nkx2.1-Cre* result in an increase in SST + CINs at the expense of fated PV + progenitors in addition to gross loss of CINs (Pai et al., 2019). Later studies implicated other important TFs as targets of the *Mafs*, including *Mef2c* (Pai et al., 2020), as a primary driver of some phenotypes; *Mef2c* was initially identified as a TF involved in PV CIN fate and development (Mayer et al., 2018) and its use as an early marker of PV-lineage CINs was hinted at by expression in early postnatal CINs (Pai et al., 2020). While the full spectrum of Wnt/beta-catenin signaling functions are still unknown in many brain cell types, including CINs, mutations in similar GSK3-beta phosphorylation sites on MAF lead to neural symptoms in humans, including seizures and intellectual disability (Niceta et al., 2015).

## Discussion

The unique roles of cellular signaling proteins in the development and function of CINs is just beginning to be uncovered. Here, we focused on three critical pathways with broad applications for many CIN subgroups. Their role in brain development and specific CIN milestones reveal distinct and shared functions that will have major implications for the development field as well as neurological/neuropsychiatric disorders caused by protein disruptions. While the mTOR, MAPK, and Wnt/Beta-catenin pathways are just some of the cellular signaling families that could modulate CIN development, future studies are likely to uncover more signaling implications. When combined with the instruction of TFs and activity-dependent maturation, it suggests the potential for complex regulation of CIN development.

An interesting finding from these initial studies is that within the core signaling families, distinct proteins can exhibit unique functions in CINs. For example, both PTEN and TSC proteins are inhibitors of the mTOR pathway, yet uniquely impact SST CIN numbers, PV axon outgrowth, PV expression and electrophysiological properties (Vogt et al., 2015; Malik et al., 2019; Haji et al., 2020). Some of these discrepancies may arise from the timing of gene deletion during development. Moreover, differential loss of PTEN or TSC can result in opposite impacts on AKT (Cantley and Neel, 1999; Huang and Manning, 2009); loss of each results in the same increased activity of mTOR, yet PTEN loss increases AKT signaling while TSC loss decreases AKT activity. Which signaling events may be required for differential CIN milestones is an important future direction.

Distinct proteins in a cascade may have common phenotypes during CIN development, as was noted for manipulations of *Nf1* and *Mek* that led to hyperactivity of the MAPK pathway and subsequent similar losses of PV + CINs (Angara et al., 2020; Holter et al., 2021). This latter observation may be critical to uncover broader impacts that a signaling family of proteins may have during CIN development or even for disorders caused by these signaling proteins. For example, syndromes caused by various mutations in RAS/MAPK signaling proteins often lead to common comorbid symptoms, including ADHD and autism (Gutmann et al., 2012; Adviento et al., 2014; Vithayathil et al., 2018). As a family, the RASopathies account for ∼1:1,000 diagnoses. The potential discovery of shared CIN phenotypes due to various protein disruptions may be key in uncovering future therapeutics to treat RASopathy symptoms and a further understanding of CIN development, each of which could impact many.

Other considerations include the possibility that each signaling protein within a family may crosstalk with other distinct family members. For example, in cancer cells it is common that the mTOR and RAS/MAPK proteins interact (Mendoza et al., 2011). Also, the mTOR activator, AKT, can phosphorylate and inhibit GSK3beta in the WNT/beta-catenin family (Cross et al., 1995). Whether these events also occur in CINs has yet to be tested but could lead to more complex considerations during the development of CINs and the impacts of each protein. Moreover, emerging data suggest that these signaling proteins may also influence some cardinal CIN TFs, unveiling another level of CIN regulation. LHX6, which is necessary for the emergence of both SST and PV expressing CINs from the MGE (Liodis et al., 2007; Zhao et al., 2008), is repressed in *Nf1* loss of function/hyperactive MAPK mutants (Angara et al., 2020). Moreover, the *Maf* TFs, which regulate SST/PV cell properties (Pai et al., 2019, 2020), are targets of GSK3beta (Rocques et al., 2007; Herath et al., 2014), suggesting that cell signaling regulation of CIN cardinal TFs may be more common than previously expected. Future studies are needed to elucidate the full extent of these regulatory cascades to understand how CIN development can be regulated at multiple levels within the cell.

## Author contributions

All authors planned and wrote the minireview and contributed to the article and approved the submitted version.
